# Supplementary material for: α-Fodrin in Cytoskeletal Organization and the Activity of Certain Key Microtubule Kinesins
Source: Genes (Basel). 2021 May 17;12(5):750. doi: 10.3390/genes12050750 (PMC8156673; doi:10.3390/genes12050750)
Supplement: Supplementary file 1 [file genes-12-00750-s001.zip › Supp table 1.pdf]

S. No.	Uniprot ID	Uniprot Name	Gene name	Peptide count	Fold change	Anova score	Mol. wt. (Daltons)	pI
**List of Upregulated proteins**								
1	Q14240	Eukaryotic initiation factor 4A-II	EIF4A2	19	2.20	0.016	46402.27	5.33
2	Q9BQE4	Selenoprotein S	SELENOS	18	2.32	0.018	21163	9.72
3	Q86Y38	Xylosyltransferase 1	XYLT1	5	2.42	0.013	107569.23	9.32
4	Q8N0W4	Neuroligin-4, X-linked;NLGN4X	NLGN4X	5	9.76	0.015	87303.62	5.74
5	P27986	Phosphatidylinositol 3-kinase regulatory subunit alpha	PIK3R1	47	44.27	0.003	83467.2	5.83
6	Q9NUA8	Zinc finger and BTB domain-containing protein 40	ZBTB40	9	8.81	0.031	138118.3	6.11
7	Q5T0N1	Cilia- and flagella-associated protein 70	CFAP70	3	166.67	0.000	125720.71	5.57
8	O75828	Carbonyl reductase [NADPH] 3	CBR3	2	6.62	0.015	30719.06	5.82
9	Q8TD47	40S ribosomal protein S4_ Y isoform 2	RPS4Y2	7	282.41	0.020	29295.42	10.1
10	O60825	6-phosphofructo-2-kinase/fructose-2_6-bisphosphatase 2	PFKFB2	1	11.90	0.013	58345.6	8.61
11	P62269	40S ribosomal protein S18	RPS18	15	2.37	0.003	17587.48	10.99
12	O00487	26S proteasome non-ATPase regulatory subunit 14	PSMD14	8	3.51	0.000	34577.04	6.06

13	Q9NXL2	Rho guanine nucleotide exchange factor 38	ARHGEF38	4	129.33	0.016	89077.53	5.87
14	P11217	Glycogen phosphorylase_ muscle form	PYGM	16	22.38	0.005	96960.86	6.57
15	Q9Y6X6	Unconventional myosin-XVI	MYO16	9	134.82	0.006	206128.82	6.37
16	Q5FWF6	Zinc finger protein 789	ZNF789	6	4.09	0.019	49984.16	9.3
17	P21127	Cyclin-dependent kinase 11B	CDK11B	3	7.70	0.025	92619.89	5.42
18	Q99958	Forkhead box protein C2	FOXC2	2	1290.67	0.000	53719.3	8.68
19	Q8TF44	C2 calcium-dependent domain-containing protein 4C	C2CD4C	22	3.37	0.026	44576.24	9.75
20	Q9H9B1	Histone-lysine N-methyltransferase EHMT1	EHMT1	6	17.84	0.003	141335.09	5.5
21	P04350	Tubulin beta-4A chain	TUBB4A	25	12.00	0.006	49585.77	4.78
22	Q2PPJ7	Ral GTPase-activating protein subunit alpha-2	RALGAPA2	13	2.24	0.011	210769.97	5.74
23	Q6UB35	Monofunctional C1-tetrahydrofolate synthase_ mitochondrial	MTHFD1L	5	3.92	0.004	102222.59	6.72
24	P51825	AF4/FMR2 family member 1	AFF1	6	4.00	0.003	131421.52	9.26
25	Q7RTR0	NACHT_ LRR and PYD domains-containing protein 9	NLRP9	4	93.21	0.004	113312.18	6.08
26	P62987	Ubiquitin-60S ribosomal protein L40	UBA52	18	7.20	0.013	8564.84	6.56
27	P26640	Valine-tRNA ligase	VARS	8	3.60	0.003	140344.82	7.52
28	P60842	Eukaryotic initiation factor 4A-I	EIF4A1	18	2.22	0.016	46022.74	5.32

S. No.	Uniprot ID	Uniprot Name	Gene name	Peptide count	Fold change	Anova score	Mol. wt. (Daltons)	pI
**List of Upregulated proteins**								
29	Q8IUR7	Armadillo repeat-containing protein 8	ARMC8	18	7.26	0.017	75378.09	6.28
30	O95644	Nuclear factor of activated T-cells_ cytoplasmic 1	NFATC1	2	535.54	0.007	101242.76	6.52
31	P0CG48	Polyubiquitin-C	UBC	18	7.21	0.013	8564.84	6.56
32	E9PAV3	Nascent polypeptide-associated complex subunit alpha_ muscle-specific form	NACA	27	3.02	0.005	205421.52	9.6
33	P51523	Zinc finger protein 84	ZNF84	2	162.58	0.018	85457.46	9.05
34	P07437	Tubulin beta chain	TUBB	26	46.20	0.009	49670.82	4.78
35	Q9UJX0	Oxidative stress-induced growth inhibitor 1	OSGIN1	2	31.57	0.016	51992.33	6.63
36	Q9P035	Very-long-chain (3R)-3-hydroxyacyl-CoA dehydratase 3	HACD3	7	3.84	0.003	43159.55	9.04
37	Q5JWF2	Guanine nucleotide-binding protein G(s) subunit alpha isoforms XLas	GNAS	22	3.37	0.026	111024.52	4.91
38	P19447	TFIIH basal transcription factor complex helicase XPB subunit	ERCC3	4	9.95	0.020	89277.7	6.83
39	Q8N8S7	Protein enabled homolog	ENAH	4	2.88	0.008	66510.26	6.51
40	P06454	Prothymosin alpha	PTMA	2	12.59	0.002	12202.96	3.66

41	Q9BZK3	Putative nascent polypeptide-associated complex subunit alpha-like protein;NACAP1	NACA4P	27	3.02	0.005	23306.03	4.53
42	Q7Z4T8	Inactive polypeptide N-acetylgalactosaminyltransferase-like protein 5	GALNTL5	5	2.50	0.013	51427.46	8.88
43	Q9H4M9	EH domain-containing protein 1	EHD1	6	25.99	0.001	60626.86	6.35
44	O60828	Polyglutamine-binding protein 1	PQBP1	3	3.44	0.013	30341.06	5.93
45	Q02790	Peptidyl-prolyl cis-trans isomerase FKBP4	FKBP4	4	3.41	0.009	51804.56	5.35
46	P45880	Voltage-dependent anion-selective channel protein 2	VDAC2	19	1.95	0.023	31435.31	7.66
47	P67809	Nuclease-sensitive element-binding protein 1	YBX1	12	2.29	0.002	35793	9.87
48	Q14146	Unhealthy ribosome biogenesis protein 2 homolog	URB2	13	5.94	0.018	170544.11	6.97
49	P78371	T-complex protein 1 subunit beta	CCT2	15	19.15	0.003	57357.02	6.02
50	P07205	Phosphoglycerate kinase 2	PGK2	15	8.45	0.015	44664.93	8.74
51	Q9UQ88	Cyclin-dependent kinase 11A	CDK11A	3	7.70	0.025	91361.58	5.28
52	Q6ZMV9	Kinesin-like protein KIF6	KIF6	11	3.62	0.017	92569.23	6.47
53	Q96DX4	RING finger and SPRY domain-containing protein 1	RSPRY1	2	6.06	0.004	62444.1	5.29
54	Q96AJ1	Clusterin-associated protein 1	CLUAP1	6	21.05	0.030	48125.05	4.68
55	O75582	Ribosomal protein S6 kinase alpha-5	RPS6KA5	4	52.76	0.009	89865.42	6.63
56	Q9HCH3	Copine-5	CPNE5	2	3.12	0.010	65733.57	5.65

S. No.	Uniprot ID	Uniprot Name	Gene name	Peptide count	Fold change	Anova score	Mol. wt. (Daltons)	pI
**List of Upregulated proteins**								
57	B0I1T2	Unconventional myosin-Ig	MYO1G	4	8.96	0.004	116442.08	8.96
58	Q0VF96	Cingulin-like protein 1	CGNL1	29	5.48	0.002	149079.19	5.51
59	P98196	Probable phospholipid-transporting ATPase IH	ATP11A	4	9.31	0.001	129755.75	6.17
60	P22102	Trifunctional purine biosynthetic protein adenosine-3	GART	5	16.58	0.020	107636	6.27
61	Q8NEZ5	F-box only protein 22	FBXO22	3	710.00	0.006	44508.36	6.61
62	O95376	E3 ubiquitin-protein ligase ARIH2	ARIH2	7	3.69	0.004	57818.86	5.4
63	Q96RY7	Intraflagellar transport protein 140 homolog	IFT140	13	2.47	0.005	165192.81	5.69
64	O43182	Rho GTPase-activating protein 6	ARHGAP6	8	3.46	0.010	105947.49	7
65	Q8TDY2	RB1-inducible coiled-coil protein 1	RB1CC1	23	2.69	0.022	183090.86	5.3
66	Q01813	ATP-dependent 6-phosphofructokinase_ platelet type	PFKP	16	4.63	0.002	85596.06	7.5
67	P08134	Rho-related GTP-binding protein RhoC	RHOC	9	5.39	0.005	21682.93	6.2
68	Q8NFZ3	Neuroligin-4_ Y-linked	NLGN4Y	5	9.76	0.015	87193.62	5.72

69	Q68D10	Protein SPT2 homolog	SPTY2D1	11	8.92	0.009	75598.69	9.79
70	Q9UPR6	Zinc finger RNA-binding protein 2	ZFR2	4	3.22	0.003	101330.37	8.76
71	P62745	Rho-related GTP-binding protein RhoB	RHOB	9	5.39	0.005	21782.92	5
72	Q9H2T7	Ran-binding protein 17;RANBP17	RANBP17	18	2.32	0.018	124244.11	6.03
73	P0CAP1	Myocardial zonula adherens protein	MYZAP	3	2.20	0.020	52247.29	5.72
74	Q92538	Golgi-specific brefeldin A-resistance guanine nucleotide exchange factor 1	GBF1	7	10.38	0.003	206445.89	5.48
75	P16152	Carbonyl reductase [NADPH] 1	CBR1	2	6.63	0.015	30243.75	8.55
76	Q92901	60S ribosomal protein L3-like	RPL3L	12	4.54	0.004	46295.98	10.45
77	Q96GQ7	Probable ATP-dependent RNA helicase DDX27	DDX27	3	21.79	0.018	89835.25	9.33
78	P0CG47	Polyubiquitin-B;UBB	UBB	18	7.21	0.013	8564.84	6.56

Supp. Table. 1

Supp. Table. 1. List of proteins upregulated upon the downregulation of α-fodrin in U-251 MG cells. The peptide count, fold change, ANOVA score, molecular wt. and pI values have also been listed.
