# Supplementary material for: α-Fodrin in Cytoskeletal Organization and the Activity of Certain Key Microtubule Kinesins
Source: Genes (Basel). 2021 May 17;12(5):750. doi: 10.3390/genes12050750 (PMC8156673; doi:10.3390/genes12050750)
Supplement: Supplementary file 1 [file genes-12-00750-s001.zip › Supp table 2.pdf]

S. No.	Uniprot ID	Uniprot Name	Gene name	Peptide count	Fold change	Anova score	Mol. wt. (Daltons)	pI
**List of Downregulated Proteins**								
1	Q86U42	Polyadenylate-binding protein 2	PABPN1	2	5.07	0.022	32617.88	5.04
2	Q03468	DNA excision repair protein ERCC-6	ERCC6	10	1.91	0.021	168416.12	8.28
3	A8K979	ERI1 exoribonuclease 2	ERI2	5	13.40	0.001	77400.64	9.12
4	Q8N4N8	Kinesin-like protein KIF2B	KIF2B	2	2.28	0.002	76253.79	8.89
5	Q9HAV7	GrpE protein homolog 1_ mitochondrial	GRPEL1	5	3.54	0.005	21336.46	6.03
6	Q13151	Heterogeneous nuclear ribonucleoprotein A0	HNRNPA0	12	3.18	0.000	30840.62	9.34
7	Q9H6S0	Probable ATP-dependent RNA helicase YTHDC2	YTHDC2	11	2.61	0.009	160248.45	8.68
8	Q8TBY8	Polyamine-modulated factor 1-binding protein 1	PMFBP1	29	3.38	0.001	117493.79	5.94
9	Q8N162	Olfactory receptor 8H2	OR8H2	1	369.66	0.001	35421.99	9.03
10	Q99445	Glycosyl-phosphatidylinositol-anchored molecule-like protein	GML	2	38.24	0.016	15918.41	6.67
11	Q14524	Sodium channel protein type 5 subunit alpha	SCN5A	3	8.46	0.002	226939.85	5.34
12	Q8N4W9	Zinc finger protein 808	ZNF808	16	1.91	0.020	104824.7	9.59

13	Q13905	Rap guanine nucleotide exchange factor 1	RAPGEF1	9	2.96	0.001	120547.62	5.64
14	Q04837	Single-stranded DNA-binding protein_ mitochondrial	SSBP1	11	2.23	0.005	15195.14	8.24
15	Q71DI3	Histone H3.2	HIST2H3A	22	2.99	0.001	15388.03	11.27
16	Q86V35	Calcium-binding protein 7;CABP7	CABP7	87	2.08	0.001	24453.07	4.56
17	Q02539	Histone H1.1	HIST1H1A	10	24.04	0.005	21710.9	10.99
18	Q9UBR2	Cathepsin Z	CTSZ	3	3.33	0.010	27149.11	5.48
19	Q4LEZ3	Alanine and arginine-rich domain-containing protein	AARD	2	3.47	0.007	17574.78	5.78
20	Q86YF9	Zinc finger protein DZIP1	DZIP1	16	2.36	0.003	98663.69	5.8
21	Q96AG4	Leucine-rich repeat-containing protein 59	LRRC59	17	2.03	0.011	34930.46	9.61
22	P19022	Cadherin-2	CDH2	3	3.69	0.002	82030.01	4.43
23	P50402	Emerin	EMD	6	2.20	0.016	28993.86	5.29
24	O15303	Metabotropic glutamate receptor 6;GRM6	GRM6	25	11.95	0.021	92772.43	7.98
25	Q8TEJ3	SH3 domain-containing RING finger protein 3	SH3RF3	4	2.56	0.005	92776.22	9.09
26	P11166	Solute carrier family 2_ facilitated glucose transporter member 1	SLC2A1	15	2.85	0.011	54083.78	8.93
27	P07355	Annexin A2	ANXA2	87	2.07	0.001	38472.85	7.56
28	Q13740	CD166 antigen	ALCAM	7	3.44	0.015	62262.86	5.71

S. No.	Uniprot ID	Uniprot Name	Gene name	Peptide count	Fold change	Anova score	Mol. wt. (Daltons)	pI
**List of Downregulated Proteins**								
29	Q9UJX4	Anaphase-promoting complex subunit 5	ANAPC5	3	1.76	0.006	85076.94	6.4
30	Q96JH7	Deubiquitinating protein VCIP135	VCPIP1	12	5.75	0.012	134320.52	6.77
31	P53985	Monocarboxylate transporter 1	SLC16A1	5	2.51	0.001	53944.15	8.91
32	P28331	NADH-ubiquinone oxidoreductase 75 kDa subunit_ mitochondrial	NDUFS1	12	8.07	0.000	76975.37	5.42
33	Q9P1V8	Sterile alpha motif domain-containing protein 15	SAMD15	5	1.79	0.006	77150.69	4.43
34	P22087	rRNA 2'-O-methyltransferase fibrillarin	FBL	13	2.67	0.005	33784.22	10.18
35	P10586	Receptor-type tyrosine-protein phosphatase F	PTPRF	2	500.00	0.000	210043.4	5.91
36	Q8WWL7	G2/mitotic-specific cyclin-B3	CCNB3	10	4.22	0.002	157916.13	6.28
37	O94901	Isoform 9 of SUN domain-containing protein 1	SUN1	11	3.14	0.003	90063.97	6.61
38	Q8N531	F-box/LRR-repeat protein 6	FBXL6	25	11.95	0.021	58588.09	9.51
39	P49642	DNA primase small subunit	PRIM1	2	23.80	0.014	49902.03	8.39
40	Q99873	Protein arginine N-methyltransferase 1	PRMT1	13	2.28	0.015	42461.64	5.18

41	Q5VYS8	Terminal uridylyltransferase 7	TUT7	31	2.64	0.003	171229.24	6.4
42	Q13224	Glutamate receptor ionotropic_ NMDA 2B	GRIN2B	6	71.53	0.008	163564.83	6.35
43	Q86UV5	Ubiquitin carboxyl-terminal hydrolase 48	USP48	14	2.42	0.010	119032.28	5.75
44	O15504	Nucleoporin-like protein 2	NUP42	2	9.41	0.011	44871.63	9.25
45	Q8WXE0	Caskin-2	CASKIN2	5	500.00	0.009	126783.3	6.63
46	P42679	Megakaryocyte-associated tyrosine-protein kinase	MATK	6	43.26	0.006	56468.94	9.01
47	P27797	Calreticulin	CALR	33	4.00	0.005	46466.37	4.29
48	Q08380	Galectin-3-binding protein	LGALS3BP	18	2.10	0.014	63276.52	5.07
49	P61313	60S ribosomal protein L15	RPL15	18	2.85	0.009	24014.88	11.62
50	P04899	Guanine nucleotide-binding protein G(i) subunit alpha-2	GNAI2	14	2.92	0.007	40319.71	5.34
51	P29279	Connective tissue growth factor	CCN2	2	2.82	0.000	35492.23	8.31
52	Q99798	Aconitate hydratase_ mitochondrial	ACO2	21	1.94	0.004	82425.78	6.85
53	Q9H583	HEAT repeat-containing protein 1	HEATR1	20	2.37	0.026	242370.23	6.11
54	P17039	Zinc finger protein 30	ZNF30	2	43.64	0.006	71417.49	9.27
55	Q9Y5L2	Hypoxia-inducible lipid droplet-associated protein	HILPDA	1	2.23	0.033	6950.1	6.71
56	P35557	Glucokinase	GCK	2	3.01	0.005	52191.43	5.1

S. No.	Uniprot ID	Uniprot Name	Gene name	Peptide count	Fold change	Anova score	Mol. wt. (Daltons)	pI
**List of Downregulated Proteins**								
57	P22626	Heterogeneous nuclear ribonucleoproteins A2/B1	HNRNPA2B1	31	1.88	0.005	37429.7	8.97
58	P31327	Carbamoyl-phosphate synthase [ammonia]_ mitochondrial	CPS1	5	21.67	0.006	160549.19	5.92
60	P17066	Heat shock 70 kDa protein 6	HSPA6	27	1.89	0.001	71028.14	5.81
61	O60759	Cytohesin-interacting protein	CYTIP	4	6.84	0.008	40009.93	6.55
62	P38159	RNA-binding motif protein_ X chromosome	RBMX	38	1.62	0.002	42331.85	10.06
63	Q14498	RNA-binding protein 39	RBM39	10	27.25	0.005	59248.39	10.1
64	Q8TEL6	Short transient receptor potential channel 4-associated protein	TRPC4AP	5	20.69	0.026	90720.86	7.62
65	Q9P258	Protein RCC2	RCC2	3	7.24	0.000	56084.52	9.02
66	Q14141	Septin-6	SEPTIN6	3	21.97	0.004	49585.46	6.25
67	Q96J84	Kin of IRRE-like protein 1	KIRREL1	3	500.00	0.004	81714.57	5.55
68	Q92843	Isoform 3 of Bcl-2-like protein 2	BCL2L2	2	5.10	0.022	20615.2	5.21
69	Q6NXT2	Histone H3.3C	H3F3C	22	3.00	0.001	15082.54	11.11

70	P11388	DNA topoisomerase 2-alpha	TOP2A	47	2.76	0.006	174385.11	8.82
71	O75526	RNA-binding motif protein_ X-linked-like-2	RBMXL2	12	22.19	0.009	42814.21	10.33
72	O14917	Protocadherin-17	PCDH17	9	9.12	0.003	124298.99	5.03
73	Q92878	DNA repair protein RAD50	RAD50	34	3.36	0.019	153892.18	6.47
74	P09525	Annexin A4	ANXA4	5	2.00	0.004	35751.52	5.84
75	Q9H963	Putative zinc finger protein 702	ZNF702P	16	1.91	0.020	15019.39	9.39
76	P13073	Cytochrome c oxidase subunit 4 isoform 1_ mitochondrial	COX4I1	7	4.48	0.004	17199.8	9.16
77	P09884	DNA polymerase alpha catalytic subunit	POLA1	4	370.41	0.003	165912.77	5.61
78	Q5TB80	Centrosomal protein of 162 kDa	CEP162	21	1.72	0.002	161943.01	5.36
79	Q96GD3	Polycomb protein SCMH1	SCMH1	25	12.13	0.021	73354	9.37
80	O94776	Metastasis-associated protein MTA2	MTA2	6	3.39	0.002	75023.09	9.7
81	P68431	Histone H3.1	HIST1H3A	12	3.00	0.001	15272.89	11.13
82	P04844	Dolichyl-diphosphooligosaccharide--protein glycosyltransferase subunit 2	RPN2	15	1.97	0.011	67054.34	5.44
83	O95197	Reticulon-3	RTN3	10	3.25	0.012	112479.86	4.84
84	P16144	Integrin beta-4	ITGB4	35	5.19	0.013	199435.85	5.69
85	Q5JSZ5	Protein PRRC2B	PRRC2B	18	2.45	0.011	242966.6	8.55

S. No.	Uniprot ID	Uniprot Name	Gene name	Peptide count	Fold change	Anova score	Mol. wt. (Daltons)	pI
**List of Downregulated Proteins**								
86	O14646	Chromodomain-helicase-DNA-binding protein 1	CHD1	8	14.61	0.011	196687.53	6.68
87	Q9Y228	TRAF3-interacting JNK-activating modulator	TRAF3IP3	8	4.82	0.012	63626.3	8.79
88	Q8IWB6	Inactive serine/threonine-protein kinase TEX14	TEX14	13	1.98	0.004	167900.67	5.03
90	Q9Y248	DNA replication complex GINS protein PSF2	GINS2	2	7.33	0.014	21427.73	5.29
91	O14561	Acyl carrier protein_ mitochondrial	NDUFAB1	2	95.37	0.009	10169.64	4.06
92	Q9ULK6	RING finger protein 150	RNF150	3	2.95	0.003	44337.31	5.12
93	Q5T9S5	Coiled-coil domain-containing protein 18	CCDC18	9	3.44	0.011	168962.39	5.52
94	P63096	Guanine nucleotide-binding protein G(i) subunit alpha-1	GNAI1	14	2.92	0.007	40229.89	5.7
95	Q9NX14	NADH dehydrogenase [ubiquinone] 1 beta subcomplex subunit 11_ mitochondrial	NDUFB11	4	12.40	0.010	14310.04	4.58
96	Q09028	Histone-binding protein RBBP4	RBBP4	12	3.03	0.017	47524.51	4.74
97	P12883	Myosin-7	MYH7	20	1.57	0.015	223097.3	5.63
98	P62805	Histone H4	HIST1H4A	25	11.96	0.021	11236.15	11.36

99	Q93045	Stathmin-2	STMN2	6	2.08	0.020	20828.05	8.4
100	Q01650	Large neutral amino acids transporter small subunit 1	SLC7A5	3	1.83	0.002	55010.24	7.9
101	A6NMY6	Putative annexin A2-like protein	ANXA2P2	87	2.08	0.001	38527.82	6.48
102	Q8TCT9	Minor histocompatibility antigen H13	HM13	8	3.19	0.016	41488.34	6
103	Q96JC4	Zinc finger protein 479	ZNF479	3	411.40	0.001	60597.86	9.21
104	P62995	Transformer-2 protein homolog beta	TRA2B	11	2.44	0.024	33534.48	11.25
105	P08758	Annexin A5	ANXA5	24	1.85	0.002	35805.58	4.93
106	P0CG00	Putative zinc finger and SCAN domain-containing protein 5D	ZSCAN5DP	1	2.62	0.003	56243.87	8.95
107	Q9NYF8	Bcl-2-associated transcription factor 1	BCLAF1	16	22.36	0.006	106122.11	9.99
108	P25787	Proteasome subunit alpha type-2	PSMA2	3	3.59	0.000	25767.39	7.12
109	Q15424	Scaffold attachment factor B1	SAFB	22	2.18	0.006	102510.38	5.32
110	Q99643	Succinate dehydrogenase cytochrome b560 subunit_ mitochondrial	SDHC	1	500.00	0.000	15347.25	9.3
111	P35637	RNA-binding protein FUS	FUS	15	5.18	0.004	53425.84	9.4
112	Q02241	Kinesin-like protein KIF23	KIF23	6	2.48	0.002	110058.81	8.76
113	Q15758	Neutral amino acid transporter B(0)	SLC1A5	10	1.75	0.017	56598.34	5.34
114	Q7L2J0	7SK snRNA methylphosphate capping enzyme	MEPCE	2	13.51	0.000	74355.24	9.62

S. No.	Uniprot ID	Uniprot Name	Gene name	Peptide count	Fold change	Anova score	Mol. wt. (Daltons)	pI
**List of Downregulated Proteins**								
115	Q9P1U1	Actin-related protein 3B	ACTR3B	4	2.50	0.008	47607.71	5.61
116	Q9C0B5	Palmitoyltransferase ZDHHC5	ZDHHC5	25	11.95	0.021	77544.73	9.17
117	Q9H560	Putative ankyrin repeat domain-containing protein 19	ANKRD19P	3	30.82	0.016	30435.89	9.15
118	Q14833	Metabotropic glutamate receptor 4;GRM4	GRM4	25	11.95	0.021	98242.4	8.99
120	P84243	Histone H3.3	H3F3A	12	3.00	0.001	15196.72	11.27
121	Q9H6S1	5-azacytidine-induced protein 2	AZI2	3	2.03	0.007	44934.82	6.15
122	Q12873	Chromodomain-helicase-DNA-binding protein 3	CHD3	30	1.99	0.005	226591.68	6.92
123	Q9BYX2	TBC1 domain family member 2A	TBC1D2	4	4.06	0.002	105413.83	6.15
124	Q92667	A-kinase anchor protein 1_ mitochondrial	AKAP1	3	371.33	0.010	93875.33	4.79
125	Q96NY7	Chloride intracellular channel protein 6	CLIC6	3	3.84	0.001	73011.52	4.29
126	Q14151	Scaffold attachment factor B2	SAFB2	31	2.47	0.009	107342.23	5.84
127	Q1MSJ5	Centrosome and spindle pole-associated protein 1	CSPP1	11	2.50	0.002	145521.98	6.37

128	Q5VVW2	GTPase-activating Rap/Ran-GAP domain-like protein 3	GARNL3	3	7.53	0.014	112852.25	7.57
129	Q8TAE8	Growth arrest and DNA damage-inducible proteins-interacting protein 1	GADD45GIP1	2	7.18	0.017	25383.83	10.03
130	Q31612	HLA class I histocompatibility antigen, B-73 alpha chain;HLA-B	HLA-B	5	5.97	0.006	37868.01	5.84
131	Q00325	Phosphate carrier protein_ mitochondrial	SLC25A3	16	2.84	0.021	34896	9.29
132	O75448	Mediator of RNA polymerase II transcription subunit 24	MED24	5	58.00	0.010	110305.27	6.51
133	Q16891	MICOS complex subunit MIC60	IMMT	29	1.77	0.007	80026.46	5.71
134	Q9Y5M8	Signal recognition particle receptor subunit beta	SRPRB	3	2.02	0.000	29702.22	9.17
135	Q9UHX1	Poly(U)-binding-splicing factor PUF60	PUF60	8	1.98	0.004	59875.47	5.19
136	Q7Z353	Highly divergent homeobox	HDX	2	1.85	0.007	77205.72	5.61
137	P43304	Glycerol-3-phosphate dehydrogenase_ mitochondrial	GPD2	24	2.19	0.020	76361.33	6.3
138	Q16695	Histone H3.1t;HIST3H3	HIST3H3	22	3.00	0.001	15377.06	11.13
139	Q96E39	RNA binding motif protein_ X-linked-like-1	RBMXL1	34	1.75	0.004	42141.58	9.9
140	P48741	Putative heat shock 70 kDa protein 7;HSPA7	HSPA7	27	1.89	0.001	40244.45	7.72
141	P51991	Heterogeneous nuclear ribonucleoprotein A3	HNRNPA3	18	6.39	0.003	39594.94	9.1
142	P02458	Collagen alpha-1(II) chain	COL2A1	4	6.55	0.002	96052.84	9.14
143	O43166	Signal-induced proliferation-associated 1-like protein 1	SIPA1L1	15	1.70	0.013	200028.5	8.4

S. No.	Uniprot ID	Uniprot Name	Gene name	Peptide count	Fold change	Anova score	Mol. wt. (Daltons)	pI
**List of Downregulated Proteins**								
144	P14210	Hepatocyte growth factor	HGF	4	2.10	0.016	53684.21	7.69
145	Q9C0K3	Actin-related protein 3C;ACTR3C	ACTR3C	4	2.49	0.008	21476.59	5.51
146	P24821	Tenascin	TNC	5	1.78	0.027	238645.74	4.79
147	Q8IWV7	E3 ubiquitin-protein ligase UBR1	UBR1	10	2.35	0.008	200079.4	5.67
148	Q99623	Prohibitin-2	PHB2	28	2.38	0.004	33165.19	9.83
150	P27824	Calnexin	CANX	32	1.70	0.004	65395.57	4.46
151	P46087	Probable 28S rRNA (cytosine(4447)-C(5))-methyltransferase	NOP2	10	1.74	0.001	89301.81	9.27
152	Q96KQ7	Histone-lysine N-methyltransferase EHMT2	EHMT2	14	1.90	0.007	132239.29	5.3
153	Q01459	Di-N-acetylchitobiase;CTBS	CTBS	21	1.70	0.002	39755.93	5.43
154	P62424	60S ribosomal protein L7a	RPL7A	21	2.35	0.003	29995.62	10.61
155	Q96K76	Ubiquitin carboxyl-terminal hydrolase 47	USP47	5	1.56	0.004	157311.32	4.97
156	P53701	Cytochrome c-type heme lyase	HCCS	5	2.78	0.014	30470.39	6.28

157	O14782	Kinesin-like protein KIF3C	KIF3C	24	2.16	0.001	89426.22	8.28
158	Q8NB50	Zinc finger protein 62 homolog	ZFP62	4	3.13	0.001	102511.07	9.24
159	Q562F6	Shugoshin 2	SGO2	18	3.56	0.015	144739.09	8.09
160	P10916	Myosin regulatory light chain 2, ventricular/cardiac muscle isoform;MYL2;	MYL2	25	11.93	0.021	18658.1	4.9
161	Q16659	Mitogen-activated protein kinase 6	MAPK6	4	2.70	0.000	82680.82	4.91
162	Q07955	Serine/arginine-rich splicing factor 1	SRSF1	20	2.24	0.002	27613.39	10.37
163	Q9NR30	Nucleolar RNA helicase 2	DDX21	35	3.56	0.006	87344.4	9.32
164	Q6DN72	Fc receptor-like protein 6	FCRL6	2	18.08	0.006	45588.98	7.24
165	P17987	T-complex protein 1 subunit alpha	TCP1	21	3.63	0.000	60343.58	5.8
166	P16949	Stathmin	STMN1	6	1.82	0.035	17171.32	5.76
167	Q7Z3Y7	Keratin_ type I cytoskeletal 28	KRT28	10	2.60	0.002	50567.39	5.33

Supp. Table. 2

Supp. Table. 2. List of proteins downregulated upon the reduced expression of α-fodrin. The peptide count, fold change, ANOVA score, molecular wt. and pI values have also been listed.
